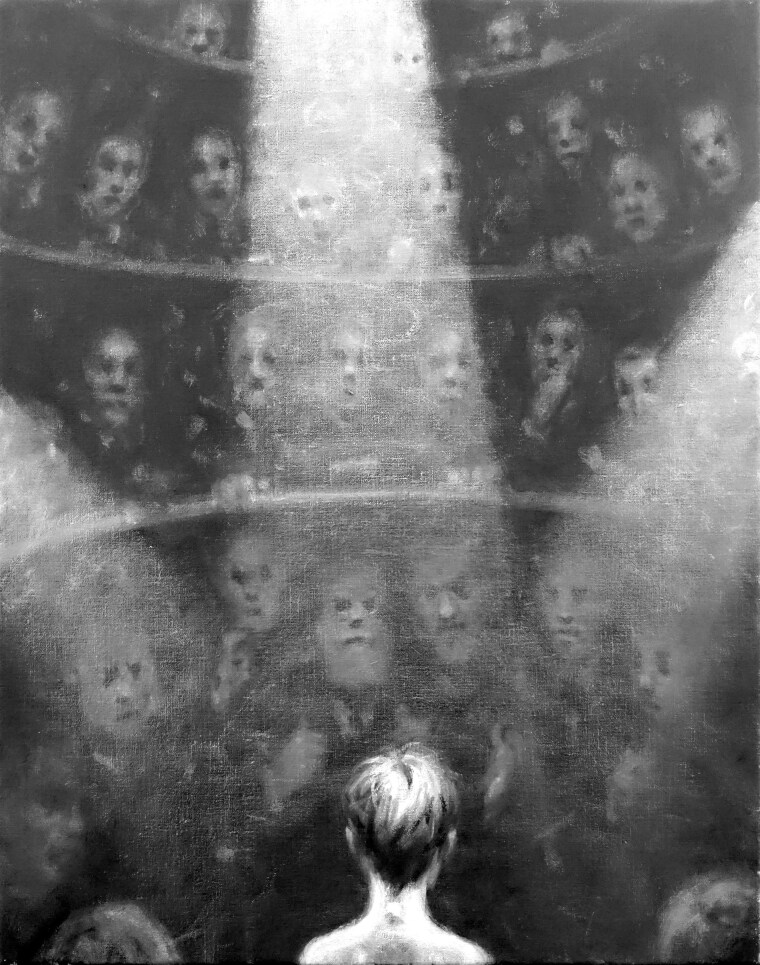# What is a neurological disease? Definition is not as simple as it might seem

**DOI:** 10.1093/brain/awaf337

**Published:** 2025-09-16

**Authors:** Simon Shorvon

**Affiliations:** Research Department of Epilepsy, UCL Queen Square Institute of Neurology, London WC1N 3BG, UK

## Abstract

Defining neurological disease is less objective than often assumed. Simon Shorvon considers how confusion between symptoms and disease, shifting criteria, unstable classifications, and complex aetiology can undermine the medical model, while labels can have profound social and personal implications the model tends to ignore.

***Examining the notion of ‘disease’ has become rather topical in recent years, triggered mainly by the remarkable recent increase in the numbers of people being diagnosed with psychiatric disorders. One reason, perhaps the predominant reason, for this rise in numbers is not that there has been any biological change but that the criteria for formulating a diagnosis have been changing.***

At the heart of this problem is the loose and malleable nature of the American Psychiatric Association’s Diagnostic and Statistical Manual of Mental Disorders (DSM), which allows diagnosis to be influenced by personal and societal as well as medical factors, resulting in, from a strictly scientific point of view, a blurring of the boundary between mental illness and mental health. Politics has played a major role (for instance, in the DSM’s handling of gender incongruence/dysphoria). ‘No more normal’ is the pointed title of an excellent book by Alastair Santhouse on this diagnostic creep, as is the recent popular science book, *The Age of Diagnosis* by Suzanne O’Sullivan. There is furthermore a large scholarly philosophical, ethical and sociology literature focusing on this and other aspects of disease-definition in its wider sense.^1-4^

On the whole, neurologists like to think that neurological disease-definition is more scientific and more rigorous than this. The belief is that neurological diagnosis relies at its core on a strictly medical model with pathology as its basis—i.e. is based on an ‘objective aetiology’ or ‘mechanism’. This, it is held, prevents diagnostic creep and diagnostic confusion. To the neurologist, organic disease has an organic causation, whereas in psychiatry, diagnosis and disease-definition make no assumptions about physical causation.

However, even when a rigid and purist medical model is applied to neurological cerebral disease-definition, there are complications which introduce subjectivity. These are the topic of this essay, and I have chosen four, illustrated mainly by the example of epilepsy although similar issues apply to other CNS (brain) conditions (n.b. I have used the term ‘disease’ here to incorporate the terms ‘disorder’, as from the perspective of the medical model, both terms are surely largely interchangeable).

First is ‘the confusion of symptom with disease’. Before 1900, the concept of a symptom was essentially synonymous with that of disease, but since then, as the sciences of pathology and histology advanced, neurological diseases became conceived as natural entities with an organic mechanistic and/or pathological basis, and symptoms as but one indication that a disease was present. However, it is easy to confuse the two. For instance, it is commonly said (after Hughlings Jackson) that the epileptic seizure is the symptom and epilepsy is the disease, but is this really the case? As science has progressed, more and more causes for epileptic seizures have been identified, and more and more pathogenic mechanisms have been found. This heterogeneity has rendered the idea that epilepsy is an entity, a ‘disease’, increasingly precarious (much as a cough or a headache are hardly ‘diseases’). Perhaps in recognition of this problem, the current formal definition of epilepsy as ‘an entity characterized by an enduring predisposition to generate seizures’, has a faintly ludicrous circularity. It would now be perhaps better to abandon the term altogether and consider epileptic seizures to be a symptom of many diseases.^5^ Another even more blatant example is that of functional neurological disorder (FND). This is an entity in which cerebral symptoms ‘of no known medical cause’ have been turned into a ‘neurological’ disorder. Even the DSM seems to recognize this incongruity, and the official term is functional neurological symptom disorder, but the word symptom has now disappeared from most neurological texts on this topic. In the past, such symptoms were considered to be disease entities such as neurasthenia, psychasthenia, hysteria, conversion disorder, Royal Free disease, but these terms have rightly fallen from use. FND seems to be a current reincarnation of this same shaky conception.

A second complication has been the effect of ‘changing criteria’ in disease-definition. In scientific medicine, changing criteria usually narrows diagnosis, for instance, by finding a new genetic or another pathological cause. However, extending criteria for no pathological or mechanistic reason can unexpectedly alter the essential nature of a disease. This has happened in migraine and to some extent in epilepsy, but autistic spectrum disorder (ASD), a disease straddling neurology and psychiatry, is a classic example. The DSM-5 in 2013 added two additional conditions into the category of ASD—pervasive development disorder not otherwise specified (PDD-NOS) and Aspergers syndrome, and since then there has been an at least 3-fold rise in the numbers of people diagnosed as having ASD, an increase likely to be largely attributable to this change in criteria. The disease traditionally referred to children, usually male, with a very severe condition comprising delayed speech, sometimes unable to talk, usually with marked cognitive deficits, repetitive behaviours and routines, and lack of ability to interact, etc. Now, much milder cases are included, and the ‘original meaning’ of the condition seems to have been almost completely lost.

A third complication relates to the tendency for frequent changes to be made in the terminology and ‘clinical classifications’ of neurological disease. Hughlings Jackson made the famous differentiation between a gardener’s (i.e. for food or ornament) and a proper (i.e. a botanical or scientific) classification, urging that only the latter is truly appropriate in scientific terms.^6^ However, in recent years, tweaks of the gardener’s type have been frequently made to the terminology and classification of some neurological diseases—epilepsy and headache are prime examples—without any scientific or mechanistic basis. Equivalent to moving deckchairs, this tinkering disconnects clinical classification schemes from the insights from science, especially molecular science, and also causes great confusion in societal setting and amongst non-specialists.

Finally, and perhaps most importantly, are complications inherent in ‘assigning aetiology’.^7^ This can be a complex task even where the underlying science is faultless. First, most cerebral diseases are multifactorial with a mixture of causes, and defining a disease by a single cause can be misleading. The historical concept of dividing neurological causation into exciting and predisposing categories was an acknowledgement of this but has now been lost. In many diseases, multiple aetiologies are present—genetic and environmental, inherent and acquired—and all may contribute significantly to the appearance of the disease. In the case in epilepsy, for instance, this is illustrated by Lennox’s famous concept of the streams filling a reservoir.^7^ Second, and also articulated by Hughlings Jackson, is that aetiology can be defined at different levels. Taking epilepsy as the example, is a seizure due to the molecular changes in a cell (the proximal cause) or the downstream effect of a lesion, for instance, a stroke or tumour (the remote cause)? This is often overlooked in clinical classifications which would look very different in these two examples. Third, as a disease develops, the underlying causes can evolve and change. The processes of epileptogenesis are a paradigmatic example of this, and whereas the initial cause may be, say, a haemorrhagic head injury, the subsequent persistence of seizures is due to entirely different processes. These problems of assigning aetiology in epilepsy are discussed in more detail elsewhere.^8^ Finally, in cases where no cause is found (the ‘idiopathic’ or cryptogenic neurological disorders), there has been a regrettable recent tendency to categorize these as ‘genetic’ even when the genetic component is either unknown or only very small. A paradigmatic example has been to change the name of ‘idiopathic generalized epilepsy’ ‘to ‘genetic generalized epilepsy’ (a change now thankfully modified), made despite the fact that no gene is known to exert any major influence in the vast majority of cases, and although a number of susceptibility genes had been discovered, they contribute only in small ways to the ‘epileptic threshold’. Already recognized environmental factors are more important, for instance, sleep deprivation, stress or alcohol intake. There is an analogy with height—a human characteristic similarly associated with a large number of inherited genetic variants but much more influenced by such factors as nutrition, childhood emotional deprivation and the ingestion of milk with hormone supplements. As David Dobbs^9^ put it, the genome of most common conditions is infected by MAGOTS (many assorted genes of tiny significance), resulting in the identification of ‘a mass of barely significant genes explaining little’. The same MAGOTS infect the epilepsy world. It would surely be better to label a disease as genetic only where genetic anomalies are the ‘overwhelming cause of the disease’ (examples include monogenic or chromosomal diseases), and not where there are only small genetic susceptibilities.

So why does this matter? Defining neurological or psychiatric brain disease is not just a dance on an academic pinhead. Mixing up symptoms and disease, changing disease criteria, and tinkering with classification and terminologies can cause confusion and perplexity, not only in medicine but also, for instance, in the law courts, in welfare legislation, in employment law, in educational provision and in litigation—in other words, unintended cultural effects. Often overlooked also is the fact that changing criteria can invalidate the conclusions of scientific study; one example from epilepsy being the finding of a link between autism and valproate teratogenicity. The data on this topic are derived mainly from a classic paper published in 2013. As autism is defined differently today, this surely renders these findings invalid as evidence of a link in current practice. Furthermore, in much scientific research, disease-definition and especially the accurate identification of aetiology provides the framework for hypothesis and investigation, and poor or inappropriate definition can prove a straitjacket impeding innovation and advance.

When it comes to chronic cerebral disorders, the organic neurological approach to diagnosis carries a further profound limitation—it ignores what to the patient and society is often the essential meaning of a ‘disease’. A disease diagnosis carries implications, sometimes grave or ominous, which are far greater than that caused simply by its medical manifestations. Making a disease diagnosis often opens a new chapter in a patient’s life, not only medically but also personally, with an impact on mood and self-esteem and on how the individual perceives him/herself and interacts with family or friends. It can also have a significant societal impact. The diagnostic label ‘epilepsy’, for instance, results in considerable stigma. The state of ‘being epileptic’ entails far more than just having brief or transient seizures, but has an impact on social interactions, social relationships, marriage, domestic life, driving, education and employment. To misquote John Berger’s famous country GP ‘a disease is less a setting for the life of its inhabitants than a curtain behind which their struggles take place’.^10^ Or, as Riese^3^ rightly pointed out, disease can be defined from other perspectives, for instance incorporating moral, ethical and ontological aspects which have nothing to do with science or medicine, and yet reflect the real impact of disease and the totality of the disease experience. Labelling a disease as genetic can have particularly dire consequences. Genetic differences assign individuals to groupings, in effect underclasses, thus rendering them vulnerable to social exclusion and coercion, to curtailing of rights and limitation of freedom. Eugenic policies have throughout history harmed many persons handicapped by neurological disorders labelled as genetic. This remains an issue today as eugenic practices persist to an extent albeit under different guises (for instance, gene editing, embryo selection, ‘eradication’ of Down syndrome by selective abortion), and where the politics of medical genetics show some similarities to those in the mid-20th century, for instance, a contradictory attitude to the disabled, the overweening power of doctors—through their specialized knowledge—to decide how genetics is incorporated into clinical decisions, and the mistaken belief that scientific and technical knowledge is ‘neutral’.

So where does this leave neurological disease definition? To rely only on a medical model has complications and is less precise than we like to imagine, and assigning aetiology, although fundamental, to the model is complex and to an extent subjective. There are also philosophical and other perspectives which are non-medical and yet add valid dimensions. Whether we like it or not, diagnoses can have a political, societal or personal impact independent of medicine, yet attempts to incorporate such aspects into disease-definition renders neurological diagnosis susceptible to the same confusion that surrounds disease-definition in psychiatry. When making diagnoses, we are indeed stepping into muddy waters.

**Figure:**